# Epidemiology and risk factors for resistance to treatment of Kawasaki disease in Cyprus

**DOI:** 10.1038/s41598-023-27694-1

**Published:** 2023-01-07

**Authors:** Maria G. Koliou, Athina Aristidou, Stella Mazeri, Elena Georgiou, Maria Agathocleous, Marianna Kousparou, Avraam Elia, Antonis Jossif

**Affiliations:** 1 Department of Paediatrics, Archbishop Makarios III Hospital, 6 Korytsas Str, Acropolis 1474, Nicosia, Cyprus; 2 Medical School, University of Cyprus, Nicosia, Cyprus; 3 Epidemiology and Public Health, Roslin Institute, Royal Dick School of Veterinary Studies, University of Edinburgh, Edinburgh, Scotland, UK; 4 Paediatric Department, Limassol General Hospital, Limassol, Cyprus; 5 Paedi Center for Specialized Paediatrics, Athalassis 178, Strovolos 2024, Nicosia, Cyprus; 6 5 Agiou Symeou Street, 2037 Strovolos, Nicosia, Cyprus

**Keywords:** Cardiology, Rheumatology

## Abstract

Kawasaki disease (KD) is one of the most common vasculitides of early childhood. There are no previous studies on KD in Cyprus. The aim of this study was to evaluate the epidemiology of KD in Cyprus, risk factors for resistance to treatment and the development of cardiac complications. This is a retrospective multicenter study of pediatric patients with KD hospitalized between January 2000 and-December 2019. The data were collected from medical records. A total of 136 patients with KD were included in the study. 83% of patients were < 5 years of age and 10% were < 6 months. Thirty patients (22%) developed coronary artery lesions. Serum sodium ≤ 133 mmol/L, albumin ≤ 3.2 g/dl, ALT ≥ 80 U/L and neutrophils percentage ≥ 80% at diagnosis, were identified as risk factors for resistance to IVIG. Clinical and epidemiological characteristics of KD in Cyprus population were similar to those reported in the literature. Although the majority of cases received appropriate treatment in time, cardiac complications still occurred.

## Introduction

Kawasaki disease (KD) is one of the most common vasculitides of early childhood and the leading cause of acquired heart disease in children in developed countries^1^. The majority of patients are younger than 5 years old. KD is very rarely encountered in adults. The disease was initially described by Tomisaku Kawasaki, a Japanese pediatrician, in 1967^2^. Japanese children have been affected more frequently having the highest incidence of the disease which seems to increase over time. Children populations in Korea and Taiwan are following, whereas the incidence in European countries and in the United States of America is much lower^3^^,^^4^. In other countries, the disease incidence remains higher in children with Japanese origin in comparison to the rest of the children population which shows that genetic factors may play an important role in the incidence of disease^4^. More than fifty years have passed since the disease was first described, yet the disease etiology remains unknown. There are many theories about the pathogenesis of KD, including an unknown infectious triggering factor^4^.

As a result of the unknown etiology of the disease, diagnosis depends on clinical criteria, which include fever, changes of lips and oral cavity, bilateral conjunctival injection, cervical lymphadenopathy, changes in the extremities and rash. The diagnosis becomes doubtful and challenging in atypical- incomplete cases where not all of the clinical criteria are met. In such cases the use of laboratory tests can be helpful in supporting the diagnosis^5^.

KD is an acute disease characterized by fever and inflammation of small and medium size arteries. Without treatment, it is a self-limited condition with an average duration of 12 days. However, about 15–25% of those who remain untreated may develop coronary artery lesions. Up to 50% of them may develop transient coronary dilation and approximately 25% may develop more persistent coronary artery abnormalities (CAA’s) such as aneurysms^6,7^. Other cardiac complications may be pericardial effusion, myocarditis and valvular regurgitation^5^*.* Moreover, in patients who receive timely treatment with intravenous immunoglobin (IVIG), only 4% develop cardiac artery abnormalities.

According to American Heart Association (AHA) guidelines, the most appropriate treatment is a single dose of IVIG (2 g/kg), concurrently with acetylsalicylic acid. A percentage of cases, despite this first-line treatment, are unresponsive. An IVIG non-responsive patient is defined as the patient whose fever persists for more than 36 h after the end of IVIG infusion^5^.

Research on factors associated with non-responsiveness to the administration of initial treatment with IVIG carried out in Japan, showed that non-responsiveness was associated with several parameters such as the young age of patient, the number of days of illness before administration of treatment and laboratory findings recorded before treatment with IVIG, such as high level of C Reactive Protein (CRP), alanine aminotransferase (ALT), aspartate aminotransferase (AST), percentage of neutrophils in full blood count (FBC), low platelet (Plt) count and serum sodium (Na) before treatment.

CAA’s is the most severe complication of KD. Factors associated with CAA’s in some studies, are the young age and the male gender, the delay of treatment beyond 10 days and non-responsiveness to the initial dose of IVIG. Studies reveal also laboratory parameters associated with the development of CAA’s such as high level of CRP^8^.

The aim of this retrospective study is to evaluate the burden, epidemiology and clinical characteristics of Kawasaki disease in the Cyprus population between 2000 and 2019. Further to this, risk factors for resistance to treatment and the development of cardiac complications were explored.

## Methods

### Data sources and collection

Medical records of patients less than 15 years of age, with complete or incomplete KD, hospitalized in Archbishop Makarios Hospital (AMH) and Limassol General Hospital between January 2000 and December 2019 were retrospectively reviewed. As Archbishop Makarios Hospital (our hospital), served as the referral center for Pediatric Cardiology services in all Cyprus, patients from other hospitals in Cyprus, were also referred to our hospital for cardiology assessment. In addition, all severe cases of KD from all Cyprus were referred to AMH for management as it also acts as the tertiary Pediatric referral center in Cyprus. All cases were categorized into complete and incomplete form, according to the American Heart Association Recommendations 2004^1^. The first day of fever was defined as the onset of disease. Initial treatment of KD was the administration of high dose IVIG (2 g/kg) together with high dose aspirin (80-100 mg/kg/d) and when the patient became afebrile for at least two to three days the dose of aspirin was reduced to 3-5 mg/kg/day. Because this study includes cases in a time frame of 20 years, treatment regimen in some cases varied. While the total dose remained the same, in the first years of the study the IVIG was administered over 2- 5 days instead of one day. Patients who remained febrile 36 h after administration of initial IVIG, were defined as IVIG non-responders. Treatment was considered as delayed when the first dose of IVIG was given after the 10th day of illness.

A database was created to include all information regarding cases. This database included demographic, clinical and laboratory as well as outcome data of the patients. Data included age, sex, season of admission, nationality, days of fever before treatment, total days of illness. It contained laboratory values before IVIG treatment (Albumin, Gamma -Glutamyl Transferase (γGT), Hemoglobin (Hb), White Blood Cell count (WBC), Percentage of Neutrophils in FBC, CRP, Erythrocyte Sedimentation Rate (ESR), Na, ALT, AST and the highest number of platelets during the course of illness.

A case of complete KD was defined as the presence of fever longer than 4 days and the presence of 4 out of the 5 clinical features that is changes of lips and oral cavity, bilateral conjunctival injection, cervical lymphadenopathy, changes in the extremities and rash. Based on the AHA guidelines a case of incomplete KD was defined as the child with fever > = 5 days and two or three of the other clinical criteria or infants with fever for at least 7 days duration without other explanation^5^.

Our group of pediatric cardiologists classified the coronary artery abnormalities according to the AHA guidelines 2004 and updated in 2017^5^ into 4 categories, depending on coronary artery Z-score which was based on assessment of populations of healthy afebrile children:

- Dilation is defined when Z-score is between 2 and 2,5, or if initially < 2 and then decrease in Z score during follow-up for at least one year.
- Small aneurysm: ≥ 2,5 to < 5
- Medium aneurysm: ≥ 5 to < 10, and absolute dimension < 8 mm
- Large or giant aneurysm: ≥ 10, or absolute dimension ≥ 8 mm

Children with KD were evaluated by Pediatric Cardiology before the initiation of treatment and prior to discharge, during the acute phase. A follow-up echo was performed between 4 to 8 weeks after diagnosis. Time of further reassessments was dependent on echocardiographic findings. In the cases that the z score of inner diameters of coronary arteries decreased by more than 1 during follow-up, then the patient was diagnosed retrospectively as a case of dilation even if the initial measurement was within normal limits.

### Ethics approval

Ethical approval was waived by the National Bioethics Committee as it was retrospective, completely anonymized and did not include any personal data.

### Statistical analysis

Data were analyzed using the R statistical software version 3.5.1^9^. Package ggplot2 was used for all plots^10^. Differences in clinical findings between complete and incomplete cases of Kawasaki disease were assessed using a chi-squared test. Blood test results between the two groups were also compared. In order to test whether there was a difference, Student T-test and U-Mann–Whitney were applied in parametric and nonparametric distributions respectively. Normality was tested by means of Shapiro–Wilk test. A *p*-value less 0.05 was considered statistically significant.

Logistic regression modeling was used to investigate factors associated with (a) resistance to intravenous immunoglobulin and (b) development of coronary artery abnormalities. Univariable analysis of all the factors against each outcome variable using Fisher’s exact test was used to select variables to be considered in the multivariable analysis. Variables with a p-value of less than 0.2 were considered for inclusion in each model. All variable combinations were considered using R package MuMIn^11^. Multivariable logistic regression models were fit using Firth’s bias reduction method from R package logistf^12^*.* Model selection was based on Second-order Akaike Information Criterion (AICc). Models with an AICc within 2 units of the lowest AICc were averaged to give the final model.

## Results

**Figure 1:**
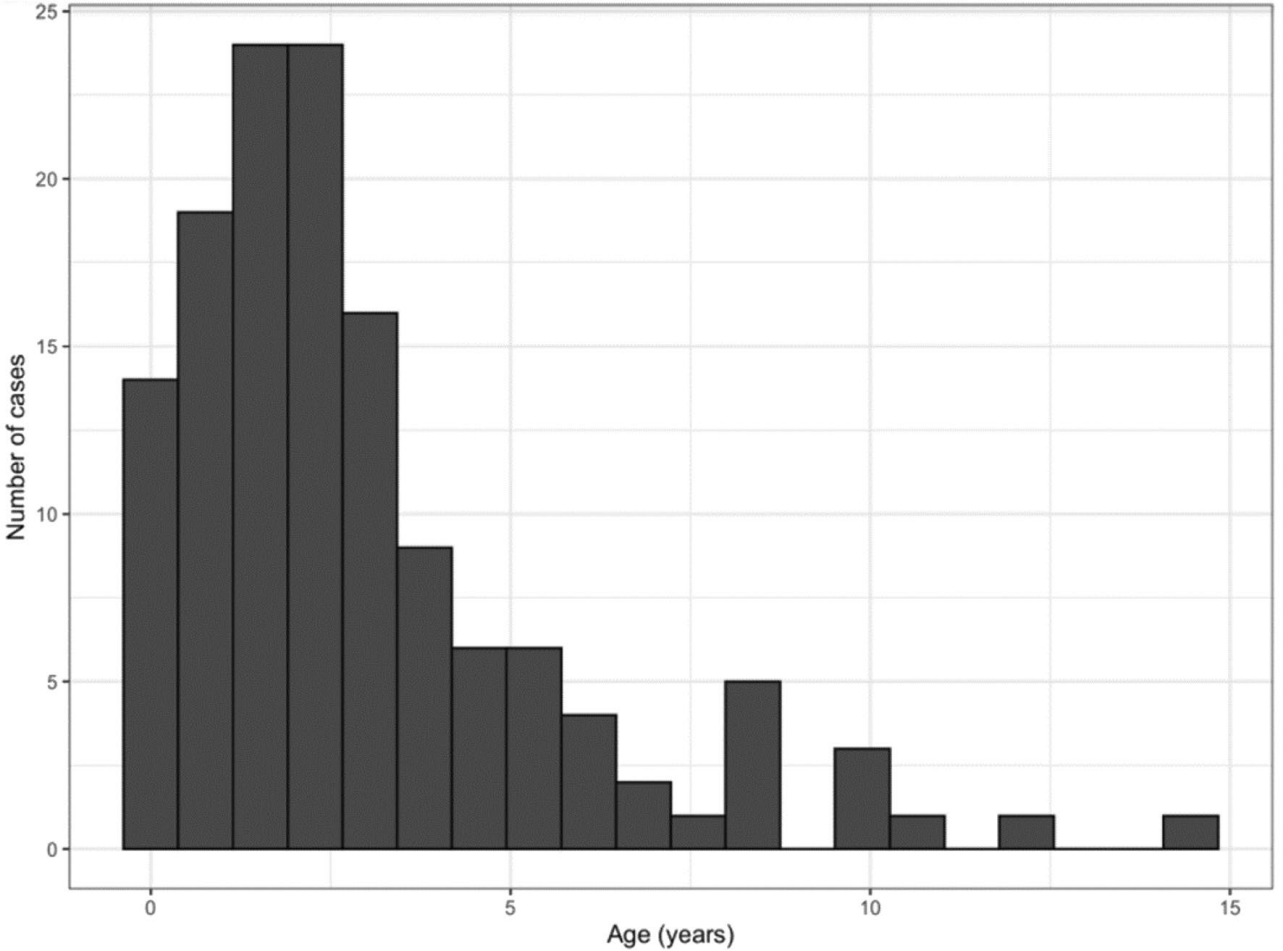
Age distribution of patients with KD.

A total of 136 patients with KD were included in the study. Male to female ratio was 1.25:1 and mean age of patients at diagnosis was 2.97 years (ranged from 0.23 to 14.67 years) and the median age 2.29 years. Eighty-three per cent of the patients were under 5 years of age. Only 32 patients (23%) were less than 12 months of age, while 14 children (10%) were younger than 6 months (Fig. 1).

The majority of the cases were diagnosed during winter and spring months (65%) (*p* = 0.0006) (Supplementary Fig. S1). The patients were mainly Greek Cypriots (88%).

Overall, 88 cases were classified as complete (69%) and 40 (31%) as incomplete Kawasaki disease. The duration of fever before administration of IVIG treatment was on average 7.2 days with a median of 6. In 17 cases diagnosis was delayed and treatment was started 10 days or more after onset of symptoms, with the longest interval between symptom onset and treatment being 22 days. A small percentage (11%) of children with KD were started on treatment less than 5 days after onset of the disease.

### Clinical characteristics

All patients presented with persistent fever for at least 5 days. Overall, changes of lips and/or oral cavity (erythema and cracking of lips, strawberry tongue, erythema of oral and pharyngeal mucosa) were the most common manifestations, seen in 113/127 patients (89%), followed by skin rash in 109/131 cases (83%). This included maculopapular, morbilliform, erythematous or urticarial rash. Bilateral bulbar conjunctival injection without exudates was seen in 103/128 patients (80%). Changes of extremities were detected in 89/126 cases (71%) in this study. Specifically, in early stages of the disease palmar or/and soles edema or/and erythema were noticed, while desquamation of digits developed in the second week of illness. Cervical lymphadenopathy was the least common clinical finding (71/124 cases -57%).

Other clinical findings which do not belong to the main criteria for diagnosis were also observed, particularly gastrointestinal (GI), musculoskeletal and central nervous system symptoms. A total of 60 out of 109 patients (55%) complained about GI symptoms. Diarrhea/abdominal pain/vomiting were the most common features in these patients. Five patients had hepatomegaly, one of them with jaundice (high level of direct bilirubin, acholic stools), and one with ascites. Musculoskeletal symptoms were present in 20/107 (19%). Arthralgia and less commonly arthritis occurred in 10 and 7 patients respectively, in various large joints. Two of these had difficulty standing up. In addition, central nervous system features developed in 15/113 patients (13.3%), extreme irritability being the most frequent symptom, followed by lethargy. Three cases, in which lumbar puncture was done, were diagnosed with aseptic meningitis. Unilateral facial nerve paresis was detected in one patient.

In Table 1 the clinical manifestations in complete vs incomplete cases of KD are compared. There were statistically significant differences in all principal criteria of KD. Changes of lips and/or oral cavity remained the most frequent clinical finding in both complete and incomplete cases.

**Table 1:** Clinical findings in patients with complete and incomplete KD (* Chi squared test).

Classical criteria	Complete KD	Incomplete KD	*P* value*
Changes of lips/oral	83(95.4%)	27(77.1%)	0.002
Bilateral conjunctival injection	78(90.7%)	21(56.8%)	< 0.001
Skin rash	82(93.2%)	23(62.2%)	< 0.001
Changes of extremities	76(86.4%)	12(34.3%)	< 0.001
Cervical lymphadenopathy	55(66.3%)	13(34.2%)	0.001
Other features			
CNS symptoms	10(13.2%)	5(14.7%)	0.771
GI symptoms	45(60.8%)	15(44.1)	0.145
Musculoskeletal	17(22.7%)	3(9.7%)	0.264

### Laboratory findings

Regarding the laboratory findings, children with complete KD had significantly lower levels of platelets before treatment in comparison to incomplete KD patients (median value 383 vs 453, *p* = 0.04) and also higher hemoglobin levels (median value 10.5 g/l vs 9.8 g/l, *p* = 0.038). In complete KD patients, liver enzymes values were higher in comparison to incomplete KD (median ALT 62 vs 22, *p* = 0.001, median AST 40 vs 29 (*p* = 0.026) (Table 2).

**Table 2:** Laboratory findings in Complete and Incomplete KD (+ U-Mann–Whitney, ++ Student T-test).

Laboratory parameter	Complete KD (variable median [IQR] n)	Incomplete KD (variable median [IQR] n)	*p*-value
Albumin g/dL	3.5 [1.7–6.8] n = 68	3.4[2.5–4.3] n = 29	0.844^+^
**ALT U/L**	**62 [8–720] n = 85**	**22[6–294] n = 40**	**0.001**^+^
**AST U/L**	**40 [16–946] n = 85**	**29.5[14–375] n = 40**	**0.026**^+^
CRP mg/dL	15 [0–40] n = 81	11.5[1–40] n = 38	0.101^+^
ESR mm/h	70.5[6–140] n = 86	77.5[16–152] n = 40	0.221^++^
**Hb g/dl**	**10.5[8–12.6] n = 81**	**9.8[5.7–12.7] n = 35**	**0.038**^++^
Highest Plts × 10^9/L	707.5[392–1375] n = 82	738.5[320–1368] n = 38	0.526^+^
**Plts before IVIG**	**383[53–1042] n = 88**	**453[21–1368] n = 40**	**0.040**^+^
Wbc × 10^9/L	15.6[1.5–40.1] n = 87	15.9[7.3–37.8] n = 40	0.734^+^

Numbers and letters in bold are referring to statistically significant values.

### Cardiological complications

A total of 56 (41.1%) patients developed some form of cardiological complication in the acute phase. As shown in Table 3, coronary artery abnormalities were the most frequent cardiological complication (22.1%). The majority of them (23/30 patients) developed transient dilations. In one of these cases the patient developed concurrently myocarditis with ventricular dysfunction. Small aneurysms were detected in four cases and medium diameter aneurysms in two patients. In two cases, the initial echo revealed a small aneurysm, and on subsequent echocardiographic investigation it further expanded, becoming in one case a giant aneurysm. Pericardial effusion was the second most common complication (18%) in acute phase.

**Table 3:** Cardiological findings in patients with KD.

Cardiological finding	Overall frequency of findings (N (%))	Acute phase (pre-treatment)	Acute phase (post-treatment)	Convalescent phase	Remote phase
Pericardial effusion	24 (17.6)				
Ventricular dysfunction	3 (2.2)				
Valvular regurgitation	7 (5.1)				
Coronary artery abnormalities	30 (22.1)	25 (18.4%)	21 (15.4%)	9 (6.6%)	5 (3.7%)
Dilation/ectasia	23 (16.9)	18	14	5	2
Small aneurysm	4 (2.9)	6	5	1	1
Medium aneurysm	2 (1.5)	1	2	3	1
Giant aneurysm	1 (0.7)	0	0	0	1
Total	56 (41.1)				

Table 3 shows the frequency of CAAs in different phases of the disease. It is evident that most of these abnormalities were detected in the acute pre-treatment phase (18.4%) while these abnormalities were becoming less frequent in the post-treatment and the convalescent phase. In 18 patients dilations were evident from the initial pre-treatment phase and they gradually resolved whereas in 5 other patients these abnormalities were not evident in the pre-treatment phase and they developed later in the post-treatment phase.

### Treatment and response

A large proportion of all the patients (108/129 -84%) responded well to the initial treatment with IVIG. Fourteen out of the 21 non-responders became afebrile after the administration of a second dose of IVIG. Five of the remaining seven patients who did not respond to two doses of IVIG responded to a third dose, while two patients required intravenous pulse of methyl prednisolone. One of these two patients additionally required treatment with infliximab, cyclosporin and tocilizumab. The median interval between onset of fever and administration of IVIG was 6 days in complete cases and 6.5 days in incomplete cases (*p*-value = 0.339).

### Risk factors for resistance to intravenous immunoglobulin

**Figure 2:**
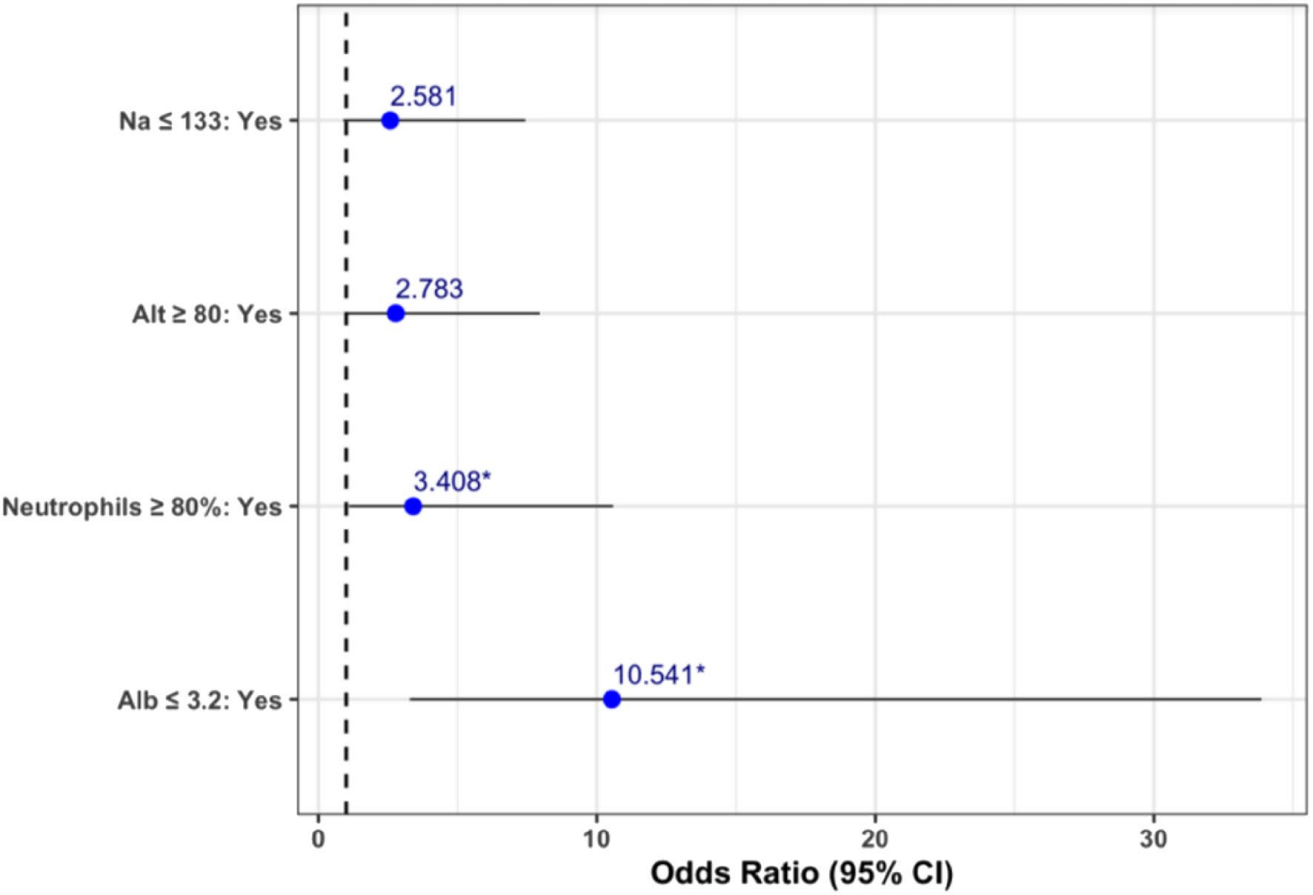
Multivariable logistic regression analysis of factors associated with IVIG non-response.

A total of 21 patients (16%) were non-responders to the initial administration of IVIG. A univariable analysis (Supplementary Table S1) was carried out in order to identify clinical and laboratory factors for resistance to IVIG. Non- responders had more commonly lower level of serum albumin and sodium, and higher level of ALT. In our population the age of patients and the day of administration of IVIG were not found to be risk factors for non-responsiveness. Multivariable regression analysis (Fig. 2) suggested that four variables were risk factors for IVIG resistance: Sodium ≤ 133 mmol/L, Albumin ≤ 3.2 g/dl, ALT ≥ 80 U/L and percentage of neutrophils ≥ 80%.

### KD and coronary abnormalities

Overall, 30 patients (22%) developed coronary artery abnormalities. Univariable logistic regression analysis (Table 4) indicated that when compared with children less than 6 months old (reference category), children between 1 and 5 years had lower odds of developing CAAs. Odds of developing CAAs in age groups 6–12 months and 60 months or older, were lower than the reference category, but these differences were not statistically significant. No significant associations between gender, delayed treatment, the total duration of fever and blood parameters such as Hb, Na, Plts and CAAs were observed in our study population (Supplementary Table S2).

**Table 4:** Univariable logistic regression of the association between age and CAAs.

Variable	Odds ratio	95% confidence interval	*P*-value
Age			
< 6 months	1.0	Reference value	
6–12 months	0.34	0.08–1.37	0.129
12–59 months	0.10	0.03–0.33	< 0.001
60 months or older	0.33	0.08–1.31	0.116

## Discussion

This is the first study on KD carried out in Cyprus using National multicenter data. The epidemiology of the disease in our country, has similar characteristics with the previous studies in other countries especially those sharing the same climatic conditions such as Spain and Greece^13,14^. There is a slight preponderance of males (male/female ratio 1.25:1), which is similar to Japan and Taiwan and also in concurrence with reports from West Asian countries, such as studies from Kuwait and Western Saudi Arabia. Exception was one study from Jordan, where it was found that the male to female ratio was much higher (3.9:1)^15–19^. Children younger than 5 years old are the most frequently affected (83% of cases) with median age of cases 2.23 years, similar to other European countries and countries in West Asia. These findings were different than Japan where the incidence rate was highest among children aged 6–11 months^15–19^. The onset of KD was more frequent in winter and spring (65%), which is consistent with findings in European reports and in neighboring countries^13,20^*.* However, in reports from some East Asian countries, such as China and Taiwan, the highest incidence rates were reported during summer. Interestingly, in Japanese and Korean population in addition to the summer peak, a second peak was noted during winter^15,19–21^.

The majority of children with KD in Cyprus presented with changes of the lips/oral cavity (89%), which is in accordance with reports from other countries^13,14^. Followed by the skin rash, which considerably varied in nature. More precisely, maculopapular was the most common form, while in two cases a petechial rash was present. Petechial rash is not described as a frequent finding in other studies. Whilst bilateral bulbar conjunctival injection without exudates was the first or second most frequently seen manifestation in some studies^13^, in our study it was the third most frequent manifestation noted (80.4%). Unilateral cervical lymphadenopathy was the least common finding (57%), which was in accordance with other studies^5^.

Diagnosis of complete and incomplete KD depends on clinical and/or laboratory criteria. The rate for incomplete cases in our population was 31%, which is consistent with the rate found in a study in the Spanish population but differs from the rate in a study from Japan where the rate of incomplete disease was only 10%^13,23^. However, the percentage of patients with incomplete KD from studies in other Asian countries, such as Shanghai and South Korea were slightly higher than our findings^20,21^. In general, the rate of incomplete KD is underestimated and is higher in infants than in older age groups^23,25^. As expected, all clinical criteria were detected at a significantly lower rate in incomplete vs complete cases. However, similar to complete cases, changes of lips and oral cavity was the most frequent clinical finding in incomplete cases. This was similar to the findings in the Spanish population^13^*.* Hemoglobin levels on admission were found to be significantly lower in incomplete vs complete cases and AST and ALT were found to be significantly higher. Platelets before administration of IVIG were found higher in incomplete vs complete cases.

The rate of responsiveness to the first dose of IVIG administered is high (84%) similar to other countries in Europe^5,14,25^. Japanese and S. Korean population had similar rate of responsiveness, whereas studies from East China and Shanghai showed an even higher rate of responsiveness, more than 90^,^^26^. Data on the same characteristic from West Asian countries are scarce. In one study from Jordan the rate of responsiveness was similar to our results^16^. Multivariable regression analysis indicated that four laboratory parameters on admission i.e. sodium ≤ 133 mmol/L, albumin ≤ 3.2 g/dl, ALT ≥ 80 U/L and the percentage of neutrophils ≥ 80% were positively associated with non-responsiveness to IVIG. There is great variation in risk factors for IVIG non-responsiveness identified in different studies. A large study in the Spanish population also revealed hypoalbuminemia and hyponatremia as risk factors for non-responsiveness. In addition to these it also revealed anemia and high procalcitonin values as risk factors^13^. In a study in San Diego USA, multivariable analysis revealed early administration of IVIG (before day 5), higher concentrations of γGT, higher % bands and lower hemoglobin, as risk factors associated with resistance to IVIG^28^.

In our population, the rate of developing CAAs was similar to results from other European countries, such as Greece and North Italy^15,27^. Our findings are also in accordance to results of studies from Korea and Shanghai, whereas in the Japanese population the development of CAAs was less common^20,21,23^. Risk factors for the development of CAAs were evaluated in our study and only young age less than 12 months was identified as a risk factor. Young age < 12 months was also identified in other studies^29^. However, some other studies have also identified other factors as risk factors for the development of CAAs such as gender, delayed treatment, the total duration of fever and blood parameters^30^.

Our study has several strengths and limitations. The retrospective nature of study means it was not possible to collect additional data relevant to Kawasaki disease and that some recorded data may have been missed. However, the records kept were very detailed, in nearly all cases, and contained most data required for the study. The study covers a 19-year period, providing one of the longest Kawasaki datasets described. The study includes children with KD who have been diagnosed since 2000, therefore the treatment regimen maybe variable in some cases, according to the existing guidelines at the time. However, the total amount of immunoglobulin administered was the same in all cases.

## Conclusions

This retrospective study was the first report on KD in Cyprus. Overall, the epidemiology of the disease, seems to be in accordance with other studies in Europe. Blood parameters such as low level of serum albumin, sodium, high level of ALT and high percentage of neutrophils in the FBC, were found as risk factors for non-responsiveness. Age was the only risk factor identified for the development of CAAs in our study.

## Supplementary Information

Supplementary Information.

## Data Availability

Most important elaborated data generated and analyzed during the study are included in this published article. Raw data will be available on request to the corresponding author.

## Abbreviations

KD: Kawasaki disease
CAAs: Coronary artery abnormalities
CRP: C reactive protein
ALT: Alanine aminotransferase
AST: Aspartate aminotransferase
FBC: Full blood count
Plt: Platelet count
Na: Serum sodium
AMH: Archbishop Makarios Hospital
γGT: Gamma -Glutamyl transferase
Hb: Hemoglobin
WBC: White blood cell count
ESR: Erythrocyte sedimentation rate
AHA: American Heart Association
AICc: Akaike information criterion
GI: Gastrointestinal
CNS: Central nervous system
IVIG: Intravenous immunoglobulin
