# Supplementary material for: Epidemiology and risk factors for resistance to treatment of Kawasaki disease in Cyprus
Source: Sci Rep. 2023 Jan 7;13:352. doi: 10.1038/s41598-023-27694-1 (PMC9825398; doi:10.1038/s41598-023-27694-1)
Supplement: Supplementary file 1 — Supplementary Information. [file 41598_2023_27694_MOESM1_ESM.docx]

**Supplementary Fig. S1**. Distribution of KD cases by month of admission to hospital


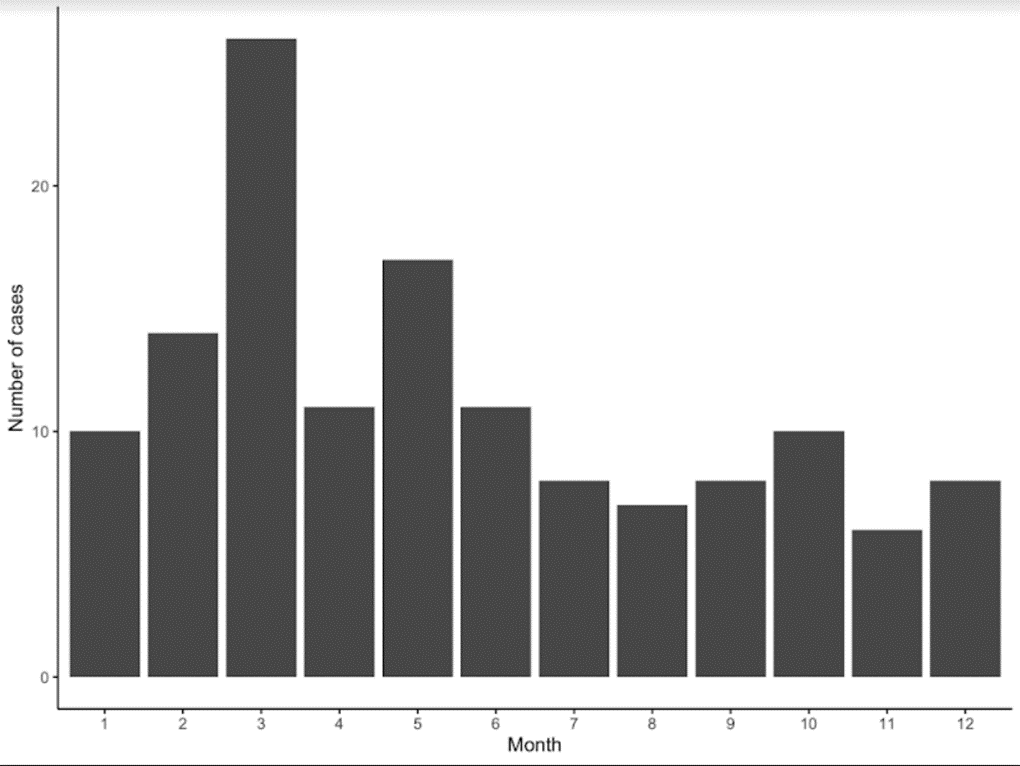


**Supplementary Table S1.** Univariable analysis for risk factors for IVIG resistance.

|  | **IVIG responsiveness (N=108)** | **IVIG resistance (N=21)** | **Total (N=129)** | **p-value** |
| --- | --- | --- | --- | --- |
| **Day of illness when treatment was given** |  |  |  | 0.490 |
| ≤4 | 10 (9.3%) | 4 (19.0%) | 14 (10.9%) |  |
| 5-10 | 82 (75.9%) | 15 (71.4%) | 97 (75.2%) |  |
| ≥10 | 15 (13.9%) | 2 (9.5%) | 17 (13.2%) |  |
| **Alb before treatment** |  |  |  | <0.001 |
| ≤ 3.2mg/dl | 15 (13.9%) | 14 (66.7%) | 29 (22.5%) |  |
| >3.2 mg/dl | 63 (58.3%) | 5 (23.8%) | 68 (52.7%) |  |
| **ESR before treatment** |  |  |  | 1.000 |
| >75mm/hr | 48 (44.4%) | 9 (42.9%) | 57 (44.2%) |  |
| ≤75mm/hr | 57 (52.8%) | 12 (57.1%) | 69 (53.5%) |  |
| **Hb before treatment** |  |  |  | 0.278 |
| <10.2 g/dL | 43 (39.8%) | 11 (52.4%) | 54 (41.9%) |  |
| ≥10.2 g/dL | 53 (49.1%) | 10 (47.6%) | 63 (48.8%) |  |
| **Neu% before treatment** |  |  |  | 0.080 |
| ≥80% | 21 (19.4%) | 9 (42.9%) | 30 (23.3%) |  |
| <80% | 85 (78.7%) | 12 (57.1%) | 97 (75.2%) |  |
| **Platelets before treatment** |  |  |  | 0.523 |
| ≤300 | 27 (25.0%) | 7 (33.3%) | 34 (26.4%) |  |
| >300 | 80 (74.1%) | 14 (66.7%) | 94 (72.9%) |  |
| **ALT before treatment** |  |  |  | 0.030 |
| ≥80 U/L | 33 (30.6%) | 13 (61.9%) | 46 (35.7%) |  |
| <80 U/L | 73 (67.6%) | 8 (38.1%) | 81 (62.8%) |  |
| **AST before treatment** |  |  |  | 0.135 |
| ≥100 U/L | 17 (15.7%) | 7 (33.3%) | 24 (18.6%) |  |
| <100 U/L | 80 (82.4%) | 14 (66.7%) | 103 (79.8%) |  |
| **Sodium before treatment** |  |  |  | 0.043 |
| ≤133 mmol/L | 30 (27.8%) | 12 (57.1%) | 42 (32.6%) |  |
| >134 mmol/L | 74 (68.5%) | 9 (42.9%) | 83 (64.3%) |  |
| **Age** |  |  |  | 0.205 |
| <6 months | 9 (8.3%) | 3 (14.3%) | 12 (9.3%) |  |
| 6-12 months | 16 (14.8%) | 0 (0.0%) | 16(12.4%) |  |
| 12-59 months | 65 (60.2%) | 14 (66.7%) | 79 (61.2%) |  |
| >60 months | 18 (16.7%) | 4 (19.0%) | 22 (17.1%) |  |
| **Sex** |  |  |  | 0.340 |
| Female | 50 (46.3%) | 7 (33.3%) | 57 (44.2%) |  |
| Male | 58 (53.7%) | 14 (66.7%) | 72 (55.8%) |  |

**Supplementary Table S2.** Univariable analysis of risk factors for development of CAAs.

|  | **Without developing CAAs N (%)** | **CAAs N (%)** | **Total N (%)** | **P-value** |
| --- | --- | --- | --- | --- |
| **Age** |  |  |  | <0.001 |
| <6 months | 5 (5.0) | 8 (25.8) | 13 (9.9) |  |
| 6-12 months | 12 (12.0) | 6 (19.4) | 18 (13.7) |  |
| 12-59 months | 69 (69.0) | 10 (32.3) | 79 (60.3) |  |
| 60 months or older | 14 (14.0) | 7 (22.6) | 21 (16.0) |  |
| **Delayed treatment** |  |  |  | 0.567 |
| No | 85 (85.0) | 24 (77.4) | 109 (83.2) |  |
| Yes | 11 (11.0) | 5 (16.1) | 16 (12.2) |  |
| **Fever duration** |  |  |  | 0.241 |
| ≤14 days | 76 (76.0) | 19 (61.3) | 95 (72.5) |  |
| >14 days | 14 (14.0) | 7 (22.6) | 21 (16.0) |  |
| **Serum Sodium** |  |  |  | 0.238 |
| <135 | 42 (42.0) | 8 (25.8) | 50 (38.2) |  |
| ≥135 | 50 (50.0) | 21 (67.7) | 71 (54.2) |  |
| **Hb** |  |  |  | 0.242 |
| ≤10,2 | 37 (37.0) | 16 (51.6) | 53 (40.5) |  |
| >10,2 | 47 (47.0) | 13 (41.9) | 60 (45.8) |  |
| **Platelets max>900 000** |  |  |  | 0.721 |
| Yes | 20 (20.0) | 7 (22.6) | 27 (20.6) |  |
| No | 71 (71.0) | 20 (64.5) | 91 (69.5) |  |
| **Sex** |  |  |  | 0.757 |
| Female | 43 (43.0) | 15 (48.4) | 58 (44.3) |  |
| Male | 56 (56.0) | 16 (51.6) | 72 (55.0) |  |
| **Season** |  |  |  | 0.931 |
| Autumn | 15 (15.0) | 4 (12.9) | 19 (14.5) |  |
| Spring | 41 (41.0) | 13 (41.9) | 54 (41.2) |  |
| Summer | 18 (18.0) | 7 (22.6) | 25 (19.1) |  |
| Winter | 26 (26.0) | 7 (22.6) | 33 (25.2) |  |
